# Down-regulation of single-stranded DNA-binding protein 1 expression induced by HCMV infection promotes lipid accumulation in cells

**DOI:** 10.1590/1414-431X20176389

**Published:** 2017-09-12

**Authors:** N. Guo, N. Zhang, L. Yan, X. Cao, F. Lv, J. Wang, Y. Wang, H. Cong

**Affiliations:** 1 Department of Cardiology, Tianjin Chest Hospital, Tianjin Medical University, Tianjin, China; 2 Department of Cardiology, Cangzhou Central Hospital, Hebei Medical University, Cangzhou, China

**Keywords:** HCMV, SSBP1, HUVEC, Lipid accumulation, LDLR

## Abstract

The objective of this study was to observe the infection of human cytomegalovirus (HCMV) to human umbilical vein endothelial cells, and its effect on the expression of single-stranded DNA-binding protein (SSBP1) and on lipid metabolism in endothelial cells. We screened the differential expression of mRNAs after HCMV infection by suppression subtractive hybridization and the expression levels of SSBP1 mRNA and protein after HCMV infection by real-time PCR and western blot. After verification of successful infection by indirect immunofluorescent staining and RT-PCR, we found a differential expression of lipid metabolism-related genes including *LDLR*, *SCARB*, *CETP*, *HMGCR*, *ApoB* and *LPL* induced by HCMV infection. The expression levels of SSBP1 mRNA and protein after HCMV infection were significantly down-regulated. Furthermore, we found that upregulation of SSBP1 inhibited the expression of atherosclerosis-associated *LDLR, SCARB, HMGCR, CETP* as well as the accumulation of lipids in the cells. The results showed that the inhibition of SSBP1 by HCMV infection promotes lipid accumulation in the cells.

## Introduction

Most of the viral infections humans encounter during their life are effectively cleared by the immune system. Examples of such viruses are influenza and respiratory syncytial virus. Not all viruses, however, are dealt with so effectively. All herpes viruses, like human cytomegalovirus (HCMV) and Epstein Barr virus, and other viruses like HIV persist after the primary infection, hiding or constantly escaping from the immune system. HCMV is a species of the Cytomegalovirus genus of viruses. It is a DNA virus that belongs to the herpes virus family type 5. It can infect any population, with 80% of infection rate before the age of 3, up to 100% of infection rate in adulthood. HCMV can easily establish latent infection in T cells and vascular endothelial tissue after primary infection in most of individuals with normal immunity. HCMV remains latent within the body throughout life but can be reactivated at any time. The virus can be activated and induce a disseminated infection with damage to a wide range of tissues and organs. HCMV is thought to be closely related to lipid metabolism disorders and plays an important role in the pathogenic mechanism of various diseases, especially atherosclerosis (AS) (1).

AS is a specific form of arteriosclerosis in which an artery wall thickens as a result of invasion and accumulation of white blood cells (foam cells) and proliferation of intimal-smooth-muscle cell creating an atheromatous (fibrofatty) plaque. The formation of an atheromatous plaque is a slow process, which develops through a complex series of cellular events occurring within the arterial wall and in response to a variety of local vascular circulating factors. The association of HCMV infection with atherogenic lesions has been well documented since Fabricant and Fabricant induced pathological changes in chickens similar to human AS using bird Marek's disease herpes virus (2). Several studies have shown that HCMV infection is one of the important pathogenic factors of AS (3,4).

HCMV may participate in AS through many ways including excessive damage to vascular endothelial cells; enhanced platelets activation from anticoagulative to coagulative state and subsequent adhesion with endothelial cells (3); promote proliferation and migration of smooth muscle cell (4); enhanced accumulation of cholesterol and cholesteryl ester in cells because of abnormal lipid metabolism (5); induced local immune or inflammatory response, among others (6). The high rate of HCMV infection in patients with AS indicates that there may be a link between HCMV infection and the body's lipid metabolism. However, the mechanism remains unclear.

Single-stranded DNA-binding protein (SSBP1) has a key role in binding with single strand DNA and repairing DNA wound during stress and infection (7–9). Therefore, in this study, we aimed to observe the infection of HCMV in human umbilical vein endothelial cells (HUVECs), and its effect on the expression of SSBP1 and on lipid metabolism in endothelial cells.

## Material and Methods

### HUVECs separation and culture

Newborn fetal umbilical cords (about 15–20 cm) were obtained under aseptic condition and washed with PBS. Fifteen milliliters of collagenase (1 mg/mL) were added to the umbilical veins at room temperature and after 15 to 20 min the digested cells were collected into a 50 mL sterile centrifuge tube by washing 2–3 times with sterile PBS. Again, the cells were washed 2–3 times with sterile PBS, centrifuged at 1000 *g* for 10 min and cultured at 37°C in 5% CO_2_. The culture media was replaced with fresh media 24 h later.

### HCMV preparation

HUVECs were maintained in DMEM (Hyclone, USA) culture media supplemented with 12% FBS (Gibco, USA), 100 U/mL penicillin and 100 μg/mL streptomycin. Human HCMV strain AD169 was preserved by Department of Microbiology, Hebei Medical University. Eighty percent confluent HUVEC cells were infected with 100 μL HCMV for 2 h at 37°C, washed two times, and then DMEM containing 3% FBS was added. The cells were gently blown and subjected to three successive freeze-thaw cycles after obvious cytopathic effects. Supernatants were harvested and tested for TCID50 followed by centrifugation to remove cell debris. The virus was aliquot and stored at –80°C until use.

### Cell culture and HCMV infection

HUVECs were cultured in a medium mixture of DMEM containing 100 U/mL penicillin, 100 μg/mL streptomycin, and 12% heat inactivated FBS and incubated at 37°C in 5% CO_2_. Passages were carried out when the cells grew into a monolayer and generation 3–8 was used in the present study. Eighty percent confluent monolayer cultures were infected with HCMV (MOI=1). The virus was allowed to incubate for 2 h at 37°C in serum-free DMEM. Thereafter, non-absorbed virus was removed by washing with DMEM twice, then the cells were cultured in fresh medium with 3% FBS until obvious cytopathic effects. HUVEC in 3% FBS DMEM2 without HCMV acted as control. HCMV infection was verified by RT-PCR amplification of HCMV major immediate-early gene (5′-GAACTCGGTAAGTCTGTTG-3′ and 5′-GTCCTCCTGCCTATGAAT-3′, 152 bp). The mixture was incubated at 95°C for 2 min and then 30 cycles of 94°C, 30 s; 44°C, 30 s; 72°C, 45 s. PCR products were identified by sequence analysis.

### Indirect immunofluorescent staining (IFA)

HUVEC were fixed by 95% ethanol and 0.1% Triton-X100. The fixed slides were blocked with a normal goat serum for 20 min and then incubated with a mouse anti-human IEpp65 monoclonal antibody (1:200) at 4°C overnight (MP Biomedicals, USA). The FITC-labeled secondary antibody (1:250) was added to the slides at 37°C for 2 h. The slides were finally mounted with mounting fluid and examined by fluorescence microscopy.

### Suppression subtractive hybridization (SSH)

Total RNA was extracted using Trizol (DingGuo, China) according to the manufacturer’s instructions and the mRNA was isolated using PolyA Ttract® mRNA Isolation System III (Z5300) Kit (Promega, USA) as described in the user manual. The quality and quantity of total RNA and mRNA were assessed using a 1% sepharose gel.

A “forward” subtractive library was constructed using the PCR-Select™ cDNA subtraction kit (Clontech, USA). The mRNA isolated from HUVECs and HCMV-infected HUVECs (24 h) were designated as “tester” and “driver”, respectively. The final PCR products were purified using PCR Product Recovery kit (DingGuo) and then cloned into the pMD19-T vector (TaKaRa, China), which was then transformed into *Escherichia coli* DH5a cells. Transformed cells were plated onto standard LB/ampicillin/X-gal/IPTG plates at 37°C for blue/white screening. A certain number of the white colonies were taken for subsequent analysis of the sequence. Colony PCR was employed to amplify the inserted cDNA fragments with pMD19-T vector universal primers M13-47 and M13-48 (M13-47:5-CGCCAGGGTTTTCCCAGTCACGAC-3, M13-48:5-AGCGGATAACAATTTCACACAGGA-3).

### Real-time PCR assay

Total RNA was extracted with the TRIzol reagent (Invitrogen, China) and reverse transcribed using the PrimeScript RT Reagent Kit (TaKaRa Biotechnology). Subsequently, real-time PCR was performed with SYBR® Premix Ex Taq™ II (TaKaRa Biotechnology) using an ABI Prism 7900 instrument (Applied Biosystems, USA). Specific primers were 5′-GGTTAGATCGTCAGGTGAGGGAG-3′ and 5′-T AAA GGAAGGTCAAGAGGAGCCA-3′ (NM_001256511, SSBP1, 173 bp); 5′-AGAGGAAATGAGAAGAAGCCCAGTA-3′ and 5′-AACCAA AGAAGGAAAGGAGCACT-3′ (NM_000527, LDLR, 454 bp); 5′- AAGAGCCCAGAGTCGGAGTTGT-3′ and 5′-GCGGCGGTGATGATGGAG-3′ (NM_ 001082959.1, SCARB, 229 bp); 5′-CAAACATTGTCACCGCCATCTAC′, and 5′-TCTTCTGTCGGACTTATCGGGC-3′ (NM_000859, HMGCR, 416 bp); 5′-ACTCCTAACCCAACTTCCACCAC-3′ and 5′-CATCATCAACCCTGAGATTATCACTC-3′ (NM_000078, CETP, 212 bp); 5′-GAAGGTCGGAGTCAACGGATTT-3′ and 5′-CTCCCTTCCTAGAATACTTAACTTAC-3′ (NM_002046, GAPDH, 224 bp).

### Western blot assay

Cell lysates were generated using RIPA lysis buffer (Dingguo) containing protease inhibitor cocktail (Roche, USA). In total, 50 μg of the cell lysates were resolved by SDS-PAGE and transferred to polyvinylidene fluoride membrane (Invitrogen). The membranes were blocked in 5% BSA, and then incubated with Polyclonal sheep anti-human SSBP1 antibodies (R&D System, USA) followed by the appropriate horseradish peroxidase-conjugated secondary antibodies. Immunoreactive bands were identified using enhanced chemiluminescence, according to the manufacturer's instructions, and quantified by densitometry.

### Plasmids and siRNA

The full coding sequence of SSBP1 was synthesized according to synthetic construct in Genebank (access No. BC093054.1) and was cloned into EcoR I and BamH I of plasmid pLenO-GFP (GenePharma, China). The constructed plasmids were verified by restriction enzyme mapping and DNA sequencing. Production of pLenO- SSBP1 was performed using the combined ratio of transfer plasmid, packaging plasmid, Env plasmid and pRSV-Rev plasmid at 4:2:1:1 by using CaCl_2_. 48 h after the virus was collected by using ultracentrifugation at 10000 *g* for 2 h. The virus pellets were then re-suspended in PBS overnight at 4°C and stored at –80°C until use. Titers were determined by fluorescence microscopy after transducing pLenO-SSBP1 into 293 T cells with different concentrations. Expression levels of SSBP1 were assayed by real-time PCR and western blot after transducing pLenO-SSBP1 into HUVECs.

SiRNA oligonucleotides for SSBP1 and negative control siRNA were purchased from Santa Cruz Biotechnology (USA). In a 6 well tissue culture plate, cells grew to 70% confluency in antibiotic-free normal growth medium supplemented with FBS. The monolayer cultures were incubated with final concentration of 10 nM siRNA or negative control to SSBP1 using siRNA Transfection Reagent (Santa Cruz Biotechnology)

### Determination of cellular total cholesterol

Cells were washed with PBS twice and were counted after SSBP1-expression or SSBP1 siRNA treatment. After counting and centrifugation at 1000 *g* for 5 min, aliquots of 200 μL cell lysates were added. By centrifugation at 1500 *g* for 10 min, supernatants were collected to measure cholesterol content according to instructions (Jixin Biotechnology, China). Briefly, cholesterol standard solution provided in the kit was appropriately diluted using cell lysate, and 1000 μL of detection reagent was added to react with 50 μL of cholesterol standard or collected supernatant. After 37°C for 10 min, absorbance at 520 nm was measured. The standard curve was prepared using cholesterol standard solution, and the concentrations of cholesterol in the sample were calculated according to the standard curve. The result was in terms of 10^6^ cells cholesterol content (μM/10^6^ cells).

### Statistical analysis

Data are reported as means±SE. Analysis was performed using SPSS10.0 for Windows (IBM, USA). Statistical significance was tested using either the *t*-test between two samples or variance among multiple samples with the chi-square test between groups. A P value of <0.05 was considered to be statistically significant.

## Results

### Infection of HUVECs by HCMV

**Figure 1.**
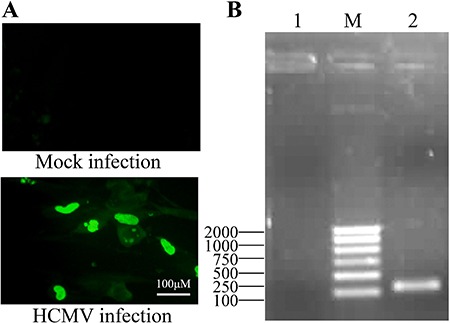
Human umbilical vein endothelial cells were successfully infected by human cytomegalovirus (HCMV). *A*, Indirect immunofluorescent staining with anti-HCMV IEpp65 monoclonal antibody was used to verify the infection. Obvious green fluorescent staining in the nuclei was observed in HCMV-infected cells (×400). *B*, HCMV major immediate-early gene was detected by real-time PCR in HCMV-infected cells.1: control; M: marker (DL2000); 2: HCMV.

The results showed the swelling of some individual cells after 24 h of infection. With prolonged incubation time, cytopathic effects became more obvious. By using IFA, green fluorescent staining in the nuclei was observed in HCMV group at 48 h (Figure 1A). At 48 h after infection, the cells were harvested and examined by RT-PCR. A 152-bp band corresponding to HCMV IE gene was amplified from HUVECs (Figure 1B). The real-time PCR products were confirmed by direct sequencing (data not shown). These results showed that HCMV successfully infected HUVECs.

### HCMV infection down-regulated the expression of SSBP1

A total of 544 insert-positive clones from the library were randomly picked and subjected to the direct sequencing with M13 forward primers. The insert sequences were manually assessed for similarities in the NCBI non-redundant (NR) protein database using BLASTX. A total of 338 ESTs were obtained, with an average length of 261 bp. The clustering analysis of those 338 ESTs yielded 58 unigenes of which 40 showed more than 95% of identity to known genes in the NR protein database (E-value<10^-5^; Table 1). The experimental results of SSH showed that there were five upregulated lipid metabolism genes including low-density lipoprotein receptor (LDLR), scavenger receptor B (SCARB), HMG-CoA reductase (HMGCR), cholesteryl ester transfer protein (CETP) and ApoB, and one down-regulated lipoprotein lipase (LPL), indicating that HCMV infection directly influenced intercellular lipid metabolism. Moreover, HCMV influenced the expression of SSBP1, which is closely related with gene stability.

**Table 1.** Transcripts that showed high homology to known genes.

Accession	*e*-value	Homologue protein
NM_001256511	1.0E-15	Homo sapiens single stranded DNA binding protein 1 (SSBP1)
NM_005000	5.0E-09	Ubiquinone oxidoreductase subunit A5 (NDUFA5)
NM_005203	1.0E-08	Homo sapiens collagen, type XIII, alpha 1 (COL13A1)
NM_016058	3.0E-12	Homo sapiens TP53RK binding protein (TPRKB)
NM_001753	2.0E-11	Homo sapiens caveolin 1 (CAV1)
NM_001010	4.0E-13	Homo sapiens ribosomal protein S6 (RPS6)
NM_052945	8.0E-21	Homo sapiens TNF receptor superfamily member 13C (TNFRSF13C)
NM_145869	6.0E-33	Homo sapiens annexin A11 (ANXA11)
NM_006844	2.0E-51	Homo sapiens ilvB acetolactate synthase like (ILVBL)
AF354444	2.0E-09	Homo sapiens IFP38 (IFP38) mRNA
NM_004333	2.0E-34	Homo sapiens B-Raf proto-oncogene, serine/threonine kinase (BRAF)
NM_139215	2.0E-14	Homo sapiens TATA-box binding protein associated factor 15 (TAF15)
NM_005895	1.0E-09	Homo sapiens golgin A3 (GOLGA3)
NM_197957	1.0E-08	Homo sapiens MYC associated factor X (MAX)
NM_138394	2.0E-11	Homo sapiens heterogeneous nuclear ribonucleoprotein L like (HNRNPLL)
NM_006087	2.0E-7	Homo sapiens tubulin beta 4A class IVa (TUBB4A)
NM_003816	3.0E-6	Homo sapiens ADAM metallopeptidase domain 9 (ADAM9)
NM_198829	6.0E-67	Homo sapiens ras-related C3 botulinum toxin substrate 1 (rho family, small GTP binding protein Rac1) (RAC1)
NM_000384	8.0E-36	Homo sapiens apolipoprotein B (APOB)
NM_003680	1.0E-47	Homo sapiens tyrosyl-tRNA synthetase (YARS)
NM_203414	1.0E-09	Homo sapiens elongator acetyltransferase complex subunit 5 (ELP5)
NM_001006605	6.0E-36	Homo sapiens family with sequence similarity 69 member A (FAM69A)
NM_173469	5.0E-06	Homo sapiens ubiquitin conjugating enzyme E2 Q2 (UBE2Q2)
NM_001634	5.0E-09	Homo sapiens adenosylmethionine decarboxylase 1 (AMD1)
NM_000237	2.0E-7	Homo sapiens lipoprotein lipase (LPL)
NR_024240	3.0E-9	Homo sapiens major histocompatibility complex, class I (HLA-I)
NM_014573	4.0E-6	Homo sapiens transmembrane protein 97 (TMEM97)
NM_014445	5.0E-10	Homo sapiens stress associated endoplasmic reticulum protein 1 (SERP1)
NM_152341	4.0E-7	Homo sapiens progestin and adipoQ receptor family member 4 (PAQR4)
NM_001001560	2.0E-8	Homo sapiens Golgi associated, gamma adaptin ear containing, ARF binding protein 1 (GGA1)
NM_000527	2.0E-18	Homo sapiens low-density lipoprotein receptor (LDLR)
NM_000859	4.0E-16	Homo sapiens 3-hydroxy-3-methylglutaryl-CoA reductase (HMGCR)
NM_001171653	2.0E-9	Homo sapiens zinc finger E-box binding homeobox 2 (ZEB2)
NM_014953	2.0E-14	Homo sapiens DIS3 homolog, exosome endoribonuclease and 3'-5' exoribonuclease (DIS3)
NM_001073	3.0E-9	Homo sapiens UDP glucuronosyltransferase family 2 member B11
NM_004800	5.0E-10	Homo sapiens transmembrane 9 superfamily member 2 (TM9SF2)
NM_001302508	2.0E-7	Homo sapiens matrix metallopeptidase 2 (MMP2)
NM_001082959	3.0E-9	Homo sapiens scavenger receptor class B member 1 (SCARB1)
NM_000078	3.0E-11	Homo sapiens cholesteryl ester transfer protein (CETP)
NM_003483	5.0E-10	Homo sapiens high mobility group AT-hook 2 (HMGA2)

**Figure 2.**
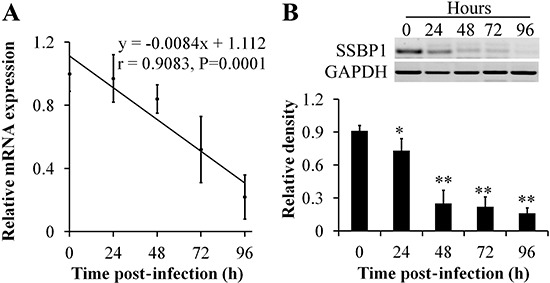
Human cytomegalovirus (HCMV) down-regulated the mRNA and protein expression of single-stranded DNA-binding protein (SSBP1). *A*, Expression levels of SSBP1 assayed by real-time PCR. *B*, Protein expression levels of SSBP1 assayed by western blot. Data are reported as means±SE, (n=4). *P<0.05 and **P<0.01 compared to time 0 (ANOVA).

Real time PCR was used to evaluate the SSBP1 expression after HCMV infection. The results showed that SSBP1 gene expression was significantly down-regulated from 24 to 96 h after infection in a time-dependent manner when compared with mock-infection group (Figure 2A). The results of western blot also showed that HCMV infection inhibited the expression of SSBP1 from 24 to 96 h (Figure 2B), indicating that HCMV infection down-regulated SSBP1 in a time-dependent manner.

### Expression of lipid metabolism-associated genes in HUVECs

**Figure 3.**
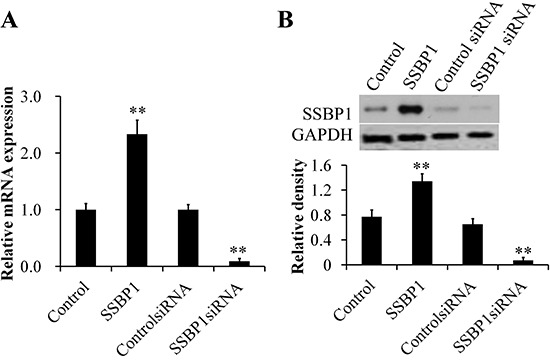
Single-stranded DNA-binding protein (SSBP1)-expression vector or siRNA were transfected into human umbilical vein endothelial cells. *A*, mRNA expression levels of SSBP1 assayed by real-time PCR. *B*, Protein expression levels of SSBP1 assayed by western blot. Data are reported as means±SE, n=4. **P<0.01 *vs* control (ANOVA).

To investigate the effects of SSBP1 on lipid mechanism, SSBP1-expression vector or siRNA were transfected into HUVECs. The efficacy of transfection was assayed by real-time PCR and western blot (Figure 3A and B).

**Figure 4.**
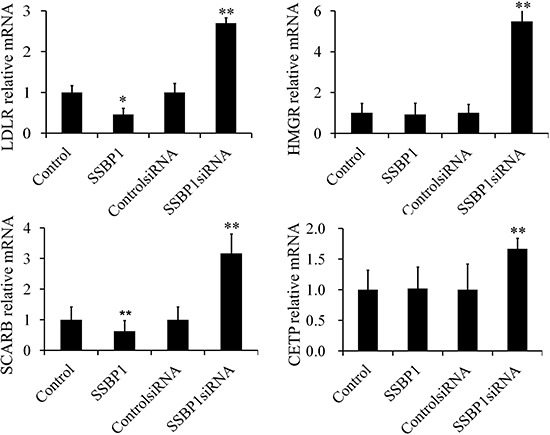
Single-stranded DNA-binding protein SSBP1 affected the expression levels of lipid metabolism-associated genes in human umbilical vein endothelial cells. The results of real-time PCR showed that over-expression of SSBP1 inhibited the expression of LDLR and SCARB, while knockdown of SSBP1 significantly promoted the mRNA expression of LDLR, HMGR, CETP and SCARB. LDLR: low-density lipoprotein receptor; SCARB: scavenger receptor B; HMGCR: HMG-CoA reductase; CETP: cholesteryl ester transfer protein. Data are reported as means±SE, n=4. *P<0.05 and **P<0.01 *vs* control (ANOVA).

Because Apo B and LPL are not mainly produced by HUVECs, we detected the changes of LDLR, HMGR, SCARB and CETP using real-time PCR, which are closely associated with the pathogenesis of AS. The results showed that, over-expression of SSBP1 inhibited the mRNA expression levels of LDLR, while knockdown of SSBP1 significantly promoted the mRNA expression levels of LDLR, HMGR and SCARB compared with control, indicating that SSBP1 inhibited AS-associated lipid uptake, synthesis and transportation (Figure 4).

### Determination of total cholesterol in HUVECs

**Figure 5.**
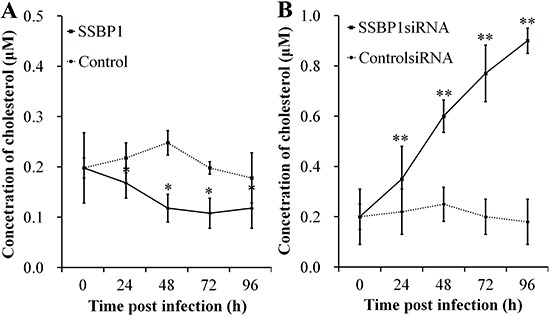
Single-stranded DNA-binding protein (SSBP1) affected the total cholesterol content. The results showed that cholesterol in the cells decreased significantly in SSBP1-expression cells (*A*) and increased significantly in SSBP1 knock-down cells in a time-dependent manner (*B*). Data are reported as means±SE, n=6. *P<0.05 and **P<0.01 (ANOVA).

HUVECs were lysed by RIPA cell lysis reagent, and the supernatants were collected for total cholesterol content assay. The results showed that from 24 to 96 h post-infection, in SSBP1 over-expressed cells, the total cholesterol contents were significantly lower than those of control (Figure 5A). However, the content of cholesterol in the cells increased significantly in SSBP1 knock-down cells in a time-dependent manner (Figure 5B), indicating that down regulation of SSBP1 induced the imbalance of cholesterol metabolism in HUVECs and led to intracellular cholesterol accumulation.

## Discussion

The formation of AS is a response to vessel wall injury. Pathogenesis of AS is an important topic in the field of cardiovascular diseases and is not yet fully understood. Two major hypotheses of AS pathogenesis are endothelial injury response and chronic inflammatory response. Chronic endothelial injury leads to accumulation of lipoproteins into the vessel walls and start an inflammatory response including adhesion of inflammatory cells (monocyte, macrophage), adhesion and activation of platelets, and finally to accumulation of lipids, extracellularly and intracellularly. In recent years, studies have shown that HCMV infection may be one of the initiating factors of AS (10). HCMV infection can induce apoptosis and dysfunction of endothelial cells *in vitro* and subsequent lipid deposition and smooth muscle cell proliferation (11,12). The experimental results of the present study showed that HCMV infection induced differential expression of lipid metabolism-related genes including LDLR, SCARB, CETP, HMGCR, ApoB and LPL. At the same time, we found that SSBP1 was also abnormally down-regulated in HCMV-infected cells.

SSBP1 binds preferentially and cooperatively to ss-DNA. It was identified as a key player in binding with single strand DNA and repairing DNA wound, especially 8-oxo-guanine and DNA double-strand breaks (7–9). Furthermore, SSBP1 has been shown to play key roles in protecting cells from proteotoxic stresses by potentiating stress-induced HSF1 transcriptional activity (13). Moreover, it has been reported that down-regulation of SSBP1 is associated with creating a metabolic state that leads to the development of obesity (14), and SSBP1 has also been identified to promote lipid accumulation in liver (15). In order to verify that HCMV can induce abnormal metabolism through the regulation of SSBP1, we constructed over expressed and silent SSBP1 vectors and examined their influence on the expression of lipid metabolism-related genes in HCMV-infected cells.

The cellular uptake of lipid is mediated by many receptors, such as very low-density lipoprotein receptor (VLDLR), low-density lipoprotein receptor protein (LDLR), scavenger receptor (16) and others. The expression levels of LDLR and SCARB are closely associated with formation of foam cells and AS by uptake of LDL and Ox-LDL, resulting in a large amount of lipid accumulation in cells. There is a close association between SSBP1 and lipid intake receptors. The study showed that the expression of LDLR and SCARB increased after SSBP1 knockdown while decreased after SSBP1 over-expression, indicating that SSBP1 inhibits uptake of lipids in the cells.

In addition to the increased cellular uptake of lipids, another important reason for accumulation of lipids in cell is increased lipid biosynthesis. A series of catalytic enzymes are involved in the synthesis of cholesterol in cells, and the most important enzyme is HMGCR, which is the rate-limiting enzyme in cholesterol synthesis and leads to abnormal cholesterol synthesis if express abnormally (17). Besides, CETP is a protein that facilitates the transport of cholesteryl esters and triglycerides between the lipoproteins membrane, which transfers cholesteryl ester from high density lipoproteins to very low-density lipoproteins in exchange for triglycerides (18). In the present study, we confirmed that expression of HMGCR and CETP increased after SSBP1 knockdown.

In order to further confirm the correlation of SSBP1 with lipid metabolism, we observed cholesterol contents in the cells after SSBP1 knockdown, which were increased significantly, indicating that SSBP1 inhibited cellular cholesterol synthesis and accumulation. However, because HUVEC could not be co-infected with HCMV and SSBP1-expressing vector successfully in the present study, whether HCMV induced abnormal lipid metabolism directly or through SSBP1 needs further study. The potential of endothelial cells to become foam cells through regulation of SSBP1 also needs further study.

In summary, we provide compelling evidence that SSBP1 knockdown promoted accumulation of lipids, whereas its over-expression restricted this process. Our findings provide insight into mechanisms and strategies for the therapeutic intervention of lipid deposition and subsequent AS.
